# Comparison of neutrophil to lymphocyte ratio and prognostic nutritional index with other clinical and molecular biomarkers for prediction of glioblastoma multiforme outcome

**DOI:** 10.1371/journal.pone.0252614

**Published:** 2021-06-17

**Authors:** Celine Garrett, Therese M. Becker, David Lynch, Joseph Po, Wei Xuan, Kieran F. Scott, Paul de Souza

**Affiliations:** 1 School of Medicine, Western Sydney University, Campbelltown, NSW, Australia; 2 Circulating Tumour Cells Group, Ingham Institute for Applied Medical Research, Liverpool, NSW, Australia; 3 School of Medicine, University of New South Wales, Kingsford, NSW, Australia; 4 School of Medicine, University of Wollongong, Wollongong, NSW, Australia; Goethe University Hospital Frankfurt, GERMANY

## Abstract

**Objective:**

Pre- and post-operative neutrophil to lymphocyte ratio (NLR) and prognostic nutritional index (PNI) and other prognostic clinicopathological variables were correlated with progression free survival (PFS) and overall survival (OS) of Glioblastoma Multiforme (GBM) patients.

**Methods:**

GBM patients (n = 87, single-centre, recruited 2013–2019) were retrospectively divided into low and high groups using literature-derived cut-offs (NLR = 5.07, PNI = 46.97). Kaplan-Meier survival curves and log rank tests assessed PFS and OS. Univariate and multivariate analyses identified PFS and OS prognosticators.

**Results:**

High vs low post-operative PNI cohort was associated with longer PFS (279 vs 136 days, *p* = 0.009), but significance was lost on multivariate analysis. Post-operative ECOG (*p* = 0.043), daily dexamethasone (*p* = 0.023) and *IDH* mutation (*p* = 0.046) were significant on multivariate analysis for PFS. High pre- and post-operative PNI were associated with improved OS (384 vs 114 days, *p* = 0.034 and 516 vs 245 days, *p* = 0.001, respectively). Low postoperative NLR correlated with OS (408 vs 249 days, *p* = 0.029). On multivariate analysis using forward selection process, extent of resection (EOR) (GTR vs biopsy, *p* = 0.004 and STR vs biopsy, *p* = 0.011), and any previous surgery (*p* = 0.014) were independent prognostic biomarkers for OS. On multivariate analysis of these latter variables with literature-derived prognostic biomarkers, EOR remained significantly associated with OS (*p* = 0.037).

**Conclusions:**

EOR, followed by having any surgery prior to GBM, are the most significant independent predictors of GBM patient’s OS. Post-operative ECOG, daily dexamethasone and *IDH* mutation are independent prognostic biomarkers for PFS. PNI may be superior to NLR. Post- vs pre-operative serum inflammatory marker levels may be associated with survival.

## Introduction

Glioblastoma Multiforme (GBM), the most common primary malignant brain tumour in adults, has a poor prognosis with an overall survival (OS) estimate of 15.5 months [1]. Biomarkers are vital in early diagnosis of high-risk phenotypes and tumour progression, and may influence treatment decisions, improving clinical outcomes [2].

Current molecular and imaging prognostic GBM biomarkers are limited by their need for tissue analysis, tumour heterogeneity, different detection methods and interobserver variability. Presently, clinical biomarkers are superior in their clinical utility, but do not reveal the pathophysiology underlying GBM. Conversely, the potential of easily-obtained serum inflammatory markers like prognostic nutritional index (PNI) and neutrophil to lymphocyte ratio (NLR) to reveal the intricate relationship between the tumour microenvironment and the systemic immune response has been demonstrated in a few studies [3, 4].

PNI reflects immunological response and nutritional status and harbors potential as a modifiable prognostic biomarker for GBM [5–7]. PNI’s prognostic capacity has been demonstrated in a variety of malignancies but its prognostic potential in GBM patients is not clear. PNI is controversial as an independent prognostic marker for OS with some studies supporting its utility [5, 6, 8], while others not [7]. Additionally, a high PNI may play a positive predictive role in adjuvant treatment efficacy [5, 8] as well as a diagnostic role in differentiating GBM from lower grade gliomas [9]. Other issues include lack of progression-free survival (PFS) data, exclusive use of preoperative PNI levels, small cohort sizes, and lack of consideration of the potential confounding effect of molecular aberrations.

NLR is indicative of systemic inflammation. The majority of GBM studies show a correlation between increased preoperative NLR levels and decreased OS on univariate analysis [3, 4, 10–12]. Other studies have attempted to create prognostic nomograms including NLR for GBM [13, 14]. Zheng et al. [9] found that NLR correlated with increasing glioma grade (*p* < 0.001) and may be useful as a means of differentiation of GBM from both low-grade gliomas and other central nervous system pathologies.

To address the current gaps in knowledge for prognostic biomarkers for GBM, specifically regarding serum inflammatory markers, this retrospective study of an Australian GBM cohort investigated whether pre- and post-operative PNI and NLR levels, in conjunction with other potential clinical and laboratory variables, correlated with PFS and OS.

## Materials and methods

### Data collection

A retrospective audit was conducted on 98 brain cancer patients at Liverpool Hospital, Australia, who had consented to have their tumour specimens stored and accessed for research in the Centre for Oncology Education and Research Translation (CONCERT) Biobank from January 2013-January 2019 (inclusive). This study was conducted in agreement with the Declaration of Helsinki; ethics approval was obtained from the Human Research Ethics Committee of the South Western Sydney Local Health District, ethics approval ID HREC/13/LPOOL/158 and HREC/12/LPOOL/459.

The inclusion criteria were: (1) histopathologically-diagnosed World Health Organization Grade IV glioma; (2) patient at Liverpool Cancer Therapy Centre; (3) age ≥ 18 years. The exclusion criteria were: (1) duplicated patient entries; (2) comorbid autoimmune, inflammatory or hematological disease; (3) no pre- or post-operative inflammatory marker levels for first tumour resection. In total, 87 patients were included in this study. For patients with infections or complications on the date of serum inflammatory marker level collection, the affected serum inflammatory marker levels were not included in the analysis. Thus, 79 and 78 patients were enrolled in the preoperative NLR and PNI cohorts, respectively, and 78 and 79 patients were enrolled into the postoperative NLR and PNI groups, respectively.

Electronic medical records (eMR) and MOSAIQ databases were interrogated for clinicopathological data, treatment regimens, pre- and post-operative serum inflammatory marker levels and survival data. Primary GBM was defined as any GBM arising de novo, whilst secondary GBM was defined as GBM developing from the progression of a lower grade glioma. Patients who received a chemotherapeutic regimen aside from conventional temozolomide (TMZ), typically due to being a participant in a clinical trial or requiring 2^nd^ line chemotherapy (bevacizumab or lomustine) following progression, were documented as having received another chemotherapy. The final date of data collection was used as the censored date for both PFS and OS. If patient records stopped prior to this date, the date of their last clinical or laboratory data entry was used as the censored date. Pre-operative data was collected on the date closest to resection within a 2-week margin and post-operative data was collected on the date furthest from resection within a 6-week margin. PNI and NLR were defined as per the equations below.


$$PNI=albumin(\frac{g}{L})+5\;X\;total\;lymphocyte\;count(\frac{10^{9}}{L})$$

$$NLR=neutrophils(\frac{10^{9}}{L})/total\;lymphocyte\;count(\frac{10^{9}}{L})$$

### Statistical methods

Statistical significance was set *a priori* at *p < 0*.*05* and 95% confidence intervals were used. All data was recorded and analyzed using SPSS software, version 25.0. The average of reported cut-off values of PNI or NLR were used to separate the patient cohort into low and high pre- and post-operative PNI and NLR groups [3–8, 11, 15–18]. Mann-Whitney U and Pearson’s Chi-squared tests were performed to assess the differences between continuous and categorical variables, respectively, and low and high PNI and NLR groups. Kaplan-Meier survival curves and log-rank tests assessed whether PFS and OS differed significantly between low and high pre- and post-operative PNI and NLR groups. A univariate analysis was conducted for PFS and OS. Variables significant (*p < 0*.*05)* in the univariate analysis for PFS were included in a multivariate cox regression analysis to determine a final model of variables significantly correlated with PFS. Variables significant (*p < 0*.*05)* in the univariate analysis for OS were included in a multivariate cox regression analysis using a forward selection process to determine the two most significant variables correlated with OS. These two variables were compared with variables that are established prognostic biomarkers within literature to determine a final model of variables significantly correlated with OS.

## Results

### Study population

Clinicopathological characteristics and treatment regimens are detailed in Table 1. Briefly, 62.1% of this cohort were males (median age 63 years). The most frequent pre- and post-operative Eastern Cooperative Oncology Group (ECOG) scores were 1 and 0, respectively (56.3% and 34.5%). Of those patients with histopathologically-analyzed specimens, 6.3% had isocitrate dehydrogenase (IDH) mutations. The most common extent of resection (EOR) was sub-total resection (STR) (49.4%), followed by gross-total resection (GTR) (35.6%) and biopsy (14.9%). The majority (77.0%) of the population received temozolomide (TMZ) with the median average dose being 220 mg/m^2^, whereas 78.2% received radiotherapy (RT) with the median total dose being 60Gy. 75.9% of patients were on daily dexamethasone, 67.8% and 58.8% were on pre- and post-operative dexamethasone on the date of data collection, respectively.

**Table 1:** Patient variables stratified by patient and tumour characteristics, treatment and comorbidities.

Variables		Frequency	Median	IQR
Patient characteristics				
Type (n = 87)				
	Primary	97.70% (n = 85)	-	-
	Secondary	2.30% (n = 2)	-	-
Gender (n = 87)				
	Male	62.10% (n = 54)	-	-
	Female	37.90% (n = 33)	-	-
BMI (n = 61)		-	27.73	23.25–31.22
Age at diagnosis (n = 87)		-	63.00	51.00–73.00
Preoperative ECOG (n = 87)				
	0	35.60% (n = 31)	-	-
	1	56.30% (n = 49)	-	-
	2	6.90% (n = 6)	-	-
	3	1.10% (n = 1)	-	-
	4	0.00% (n = 0)	-	-
Postoperative ECOG (n = 87)				
	0	34.50% (n = 30)	-	-
	1	32.30% (n = 28)	-	-
	2	18.40% (n = 16)	-	-
	3	11.50% (n = 10)	-	-
	4	3.40% (n = 3)	-	-
**Tumour characteristics**				
IDH mutation (n = 79)		6.30% (n = 5)	-	-
MGMT methylation (n = 40)		57.50% (n = 23)	-	-
EGFR (n = 25)				
	EGFR mutation	64.00% (n = 16)	-	-
	EGFRvIII	8.00% (n = 2)	-	-
Ki67 (n = 68)		-	20.00	10.00–30.00
GFAP mutation (n = 56)		98.20% (n = 55)	-	-
**Treatment**				
EOR (n = 87)				
	Biopsy	14.90% (n = 13)	-	-
	STR	49.40% (n = 43)	-	-
	GTR	35.60% (n = 31)	-	-
Temozolomide (n = 87)		77.00% (n = 67)	-	-
Temozolomide average dose (n = 70)		-	220.00	172.48–296.25
Radiotherapy(n = 87)		78.20% (n = 68)	-	-
Radiotherapy total dose (n = 70)		-	60.00	40.05–60.00
Received another CT (n = 87)		37.90% (n = 33)	-	-
Dexamethasone (n = 87)		75.90% (n = 66)	-	-
Preoperative dexamethasone (n = 87)		67.80% (n = 59)		
Postoperative dexamethasone (n = 85)		58.80% (n = 50)		
Anti-convulsants (n = 87)		63.20% (n = 55)	-	-
**Comorbidities (n = 87)**				
Neurological condition		9.20% (n = 8)	-	-
Dyslipidaemia/Hypercholesterolaemia		27.60% (n = 24)	-	-
Type II diabetes		18.40% (n = 16)	-	-
Hypertension		39.10% (n = 34)	-	-
Depression		8.00% (n = 7)	-	-
Other cancer		10.30% (n = 9)	-	-
Previous surgery		26.40% (n = 23)	-	-

BMI = body mass index, ECOG = Eastern Cooperative Oncology Group, IDH = isocitrate dehydrogenase, MGMT = O^6^methylguanine DNA methyltransferase, EGFR = epidermal growth factor receptor, GFAP = glial fibrillary acidic protein, EOR = extent of resection, STR = subtotal resection, GTR = gross total resection.

### Clinicopathological and treatment variables and NLR and PNI

The cut-off values, averaged from the literature, for NLR and PNI were 5.07 and 46.97, respectively. The median pre- and post-operative NLRs were 6.38 and 3.32, respectively. The median pre- and post-operative PNIs were 48.50 and 48.00, respectively. The distribution of patients between low and high pre- and post-operative NLR and PNI groups are displayed in Table 2. For preoperative NLR, the presence of a comorbid neurological condition was significantly different between low and high groups (*p* = 0.021), whilst for postoperative NLR, *Ki67* and postoperative dexamethasone administration differed appreciably (*p* = 0.037 and *p* = 0.000, respectively). For preoperative PNI, RT total dose and EOR were different between low and high groups (*p* = 0.010 and *p* = 0.008, respectively). However, for postoperative PNI, mutant IDH, the receival of TMZ, RT and postoperative dexamethasone as well as a patient’s postoperative ECOG score differed significantly between low and high groups (*p* = 0.028, *p* = 0.004, *p* = 0.049, *p* = 0.008 and *p* = 0.007, respectively).

**Table 2:** Low and high NLR and PNI group frequencies.

	NLR		PNI	
	Low (<5.07)	High (>5.07)	Low (<46.97)	High (>46.97)
Preoperative	44.30% (n = 35)	55.70% (n = 44)	32.10% (n = 25)	67.90% (n = 53)
Postoperative	74.40% (n = 58)	25.60% (n = 20)	46.80% (n = 37)	53.20% (n = 42)

NLR = neutrophil to lymphocyte ratio, PNI = prognostic nutritional index.

### Survival analysis as stratified by low and high NLR and PNI

**Fig 1:**
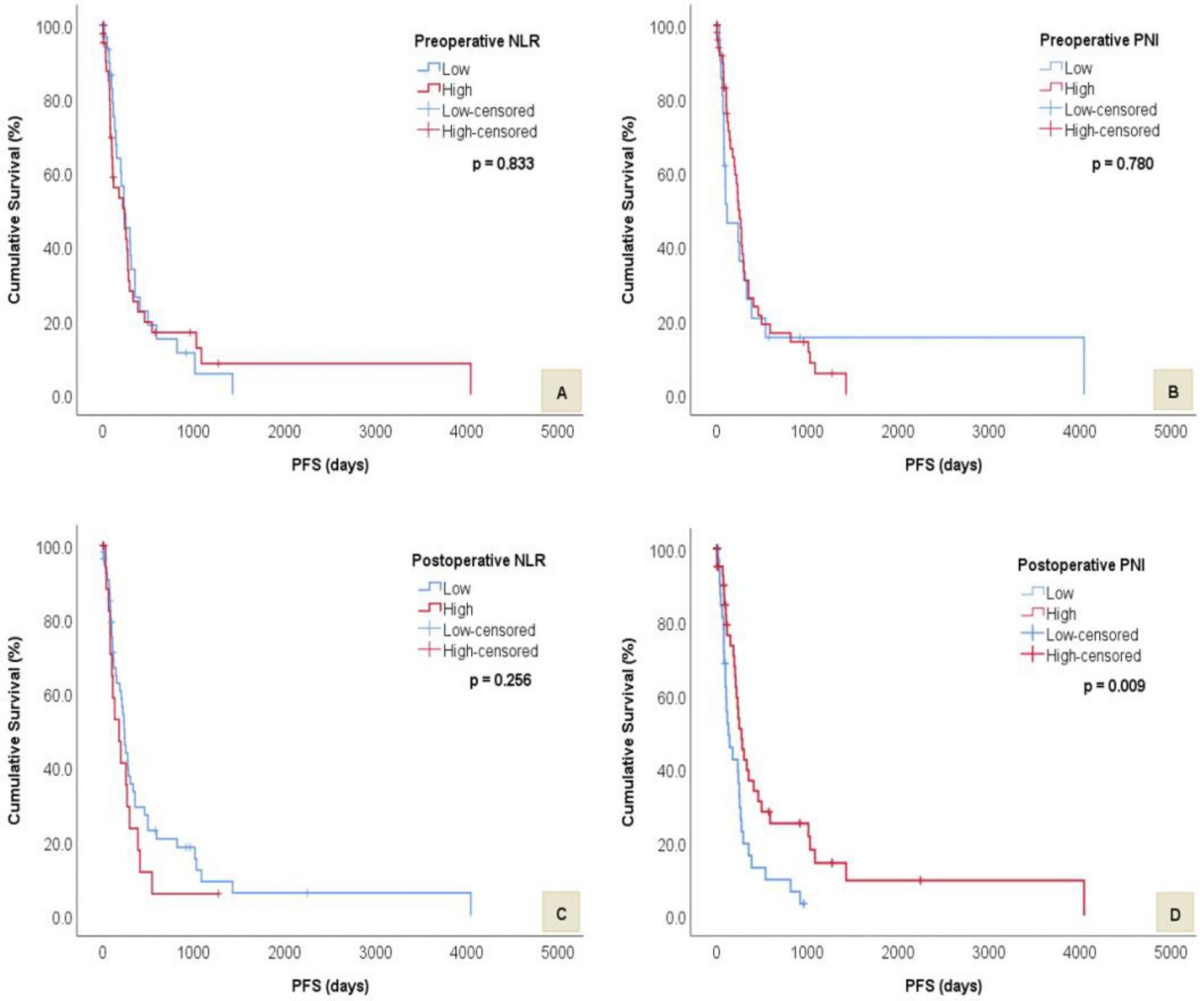
Kaplan-Meier survival curves and log rank tests showing differences in PFS between low and high preoperative and postoperative NLR and PNI groups. A) Difference in PFS between low and high preoperative NLR. B) Difference in PFS between low and high preoperative PNI. C) Difference in PFS between low and high postoperative NLR. D) Difference in PFS between low and high postoperative PNI.

**Fig 2:**
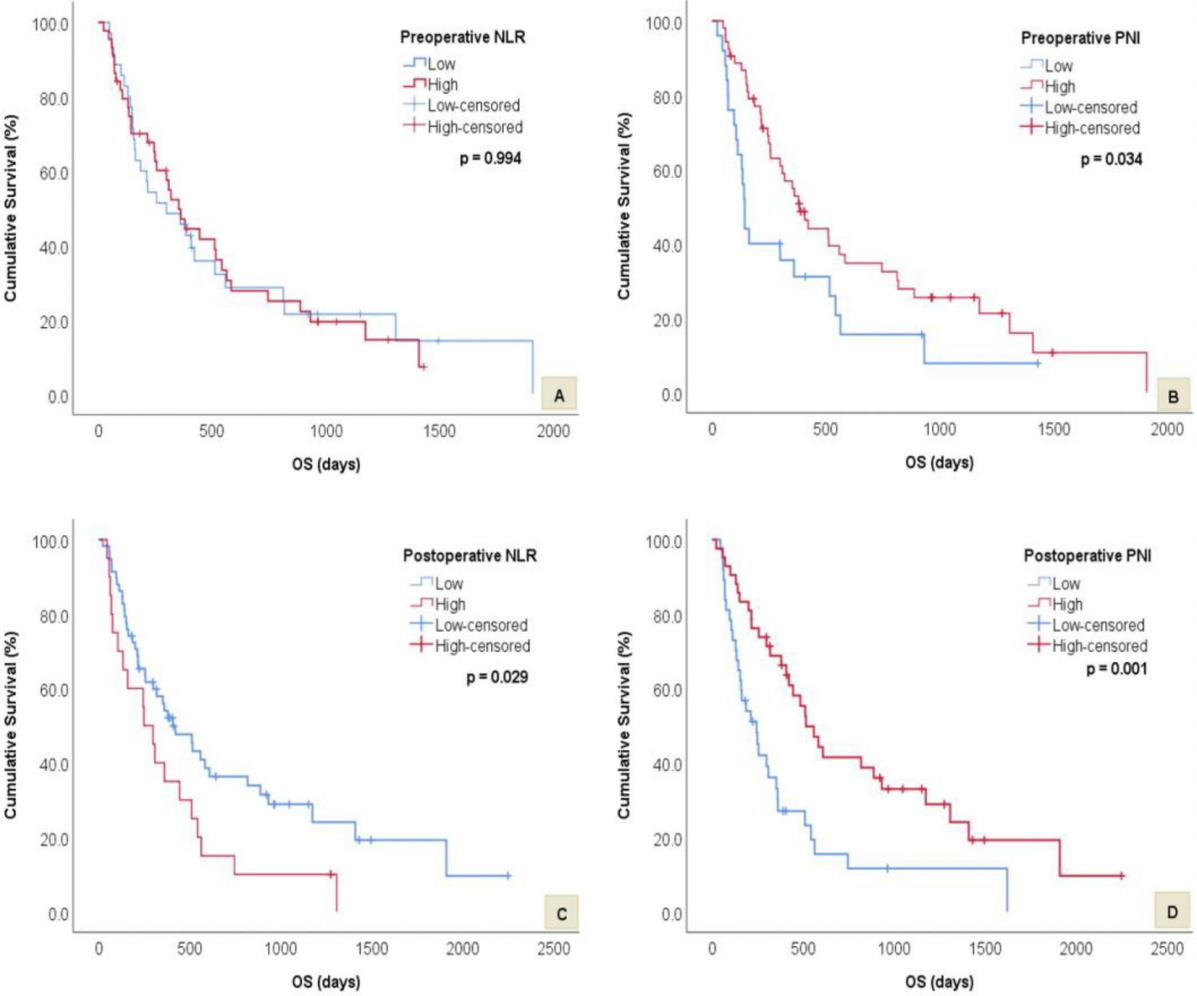
Kaplan-Meier survival curves and log rank tests showing differences in OS between low and high preoperative and postoperative NLR and PNI groups. A) Difference in OS between low and high preoperative NLR. B) Difference in OS between low and high preoperative PNI. C) Difference in OS between low and high postoperative NLR. D) Difference in OS between low and high postoperative PNI.

The median PFS and OS was 183 and 319 days, respectively. Median PFS was significantly worse for patients with low postoperative PNI (136 vs 279 days, *p* = 0.009). However, there was no significant difference in median PFS times between low and high preoperative PNI (120 vs 246 days, *p* = 0.780), preoperative NLR (239 vs 244 days, *p* = 0.833) and postoperative NLR (244 vs 183 days, *p* = 0.256) groups. Median OS was significantly shorter in patients with high postoperative NLR (249 vs 408 days, *p* = 0.029), low preoperative PNI (114 vs 384 days, *p* = 0.034) and low postoperative PNI (245 vs 516 days, *p* = 0.001). There was no significant difference in median OS times between low and high preoperative NLR groups (299 vs 353 days, *p* = 0.994). Figs 1 and 2 display the above survival results for PFS and OS respectively.

### Univariate and multivariate analyses

Significant variables associated with PFS on univariate and multivariate analysis are highlighted in Table 3. Of note, only *IDH* mutation, daily dexamethasone and postoperative ECOG score (1 vs 0) remained significant on multivariate analysis for PFS. Significant variables associated with OS on univariate analysis and multivariate analysis with forward selection process are also displayed in Table 3. The results of univariate and multivariate analysis on all variables (both significant and insignificant) are provided in a S1 Table. When the most statistically significant factors affecting OS on multivariate analysis with forward selection process (greater EOR and previous surgery) were compared to established prognostic biomarkers within literature, EOR remained significant whilst previous surgery did not (Table 4).

**Table 3:** Univariate and multivariate analyses for PFS and OS.

			Univariate analysis						Multivariate analysis					
			HR		95% CI		P value*		HR		95% CI		P value	
Variables			PFS	OS	PFS	OS	PFS	OS	PFS	OS†	PFS	OS†	PFS	OS†
Gender			0.532	0.670	0.315–0.900	0.408–1.098	**0.019**	0.112	0.722	-	0.324–1.611	-	0.427	-
BMI			0.968	0.960	0.931–1.006	0.925–0.996	0.098	**0.028**	-	-	-	-	-	0.269
IDH mutation			0.186	0.037	0.045–0.770	0.001–1.008	**0.020**	0.051	0.195	-	0.039–0.970	-	**0.046**	-
Temozolomide average dose			0.996	0.995	0.992–0.999	0.991–0.999	**0.020**	**0.018**	0.999	-	0.994–1.004	-	0.690	0.586
Radiotherapy			0.094	0.101	0.038–0.228	0.050–0.203	**<0.001**	**<0.001**	0.523	-	0.062–4.425	-	0.552	0.456
Radiotherapy total dose			0.995	0.972	0.971–1.020	0.948–0.997	0.717	**0.029**	-	-	-	-	-	-
Dexamethasone			2.643	2.536	1.419–4.922	1.336–4.816	**0.002**	**0.004**	3.599	-	1.197–10.819	-	**0.023**	0.231
Previous surgery			1.838	2.065	1.045–3.231	1.192–3.575	**0.035**	**0.010**	1.948	2.825	0.874–4.345	1.234–6.466	0.103	**0.014**
Age at diagnosis			1.019	1.026	1.002–1.036	1.008–1.045	**0.029**	**0.006**	0.987	-	0.959–1.016	-	0.378	0.530
EOR														
	STR vs biopsy		0.252	0.288	0.112–0.567	0.146–0.567	**0.001**	**<0.001**	0.408	0.205	0.102–1.628	0.061–0.697	0.204	**0.011**
	GTR vs biopsy		0.111	0.130	0.046–0.267	0.061–0.280	**<0.001**	**<0.001**	0.325	0.145	0.071–1.490	0.039–0.539	0.148	**0.004**
Preoperative														
	PNI		0.922	0.566	0.522–1.629	0.332–0.964	0.780	**0.036**	-	-	-	-	-	0.369
Postoperative														
	ECOG													
		1 vs 0	1.260	1.787	0.710–2.235	0.961–3.322	0.430	0.066	0.417	-	0.179–0.973	-	**0.043**	0.115
		2 vs 0	1.739	3.057	0.845–3.578	1.522–6.141	0.133	**0.002**	0.645	-	0.211–1.975	-	0.443	0.797
		3 vs 0	3.795	5.471	1.597–9.023	2.278–13.135	**0.003**	**<0.001**	0.900	-	0.198–4.090	-	0.892	0.056
		4 vs 0	0.000	9.587	0.000–0.000	2.079–44.208	0.982	**0.004**	-	-	-	-	-	0.057
Dexamethasone			1.806	2.581	1.092–2.985	1.525–4.370	**0.021**	**<0.001**	0.968	-	0.477–1.965	-	0.928	0.140
	NLR		1.395	1.832	0.783–2.484	1.054–3.185	0.258	**0.032**	-	-	-	-	-	0.179
	PNI		0.501	0.436	0.296–0.846	0.259–0.735	**0.010**	**0.002**	0.804	-	0.390–1.659	-	0.555	0.151

IDH = isocitrate dehydrogenase, EOR = extent of resection, STR = subtotal resection, GTR = gross total resection, ECOG = Eastern Cooperative Oncology Group, NLR = neutrophil to lymphocyte ratio, PNI = prognostic nutritional index

*P values in bold are significant,

†Results from multivariate analysis with forward selection process Ω.

**Table 4:** Multivariate analysis comparing established prognostic biomarkers from the literature to variables identified as significant in the multivariate analysis, utilising a forward selection strategy for OS.

Variable			HR	95% CI	P value*
EOR			0.347	0.129–0.936	**0.037**
Previous surgery			2.091	0.689–6.344	0.193
Age at diagnosis			1.008	0.969–1.049	0.692
IDH mutation			0.000	0.000–0.000	0.975
MGMT methylation			0.507	0.187–1.377	0.183
ECOG					
	Preoperative				
		1 vs 0	0.827	0.265–2.580	0.743
	Postoperative				
		1 vs 0	1.055	0.394–2.825	0.916
		2 vs 0	0.587	0.172–2.004	0.395
		3 vs 0	0.000	0.000–0.000	0.990

EOR = extent of resection, IDH = isocitrate dehydrogenase, MGMT = O^6^methylguanine DNA methyltransferase, ECOG = Eastern Cooperative Oncology Group.

*P values in bold are significant.

## Discussion

In general, studies of NLR and PNI in GBM patients have produced conflicting results. We found that preoperative PNI was associated with OS on univariate but not multivariate analysis whilst earlier studies found it to be a statistically significant prognostic biomarker on both univariate and multivariate analysis [5, 6, 8]. Zhou et al. [5] found that PNI < 44.4 resulted in shorter median OS (270 vs 375 days, *p* = 0.013) in comparison to PNI > 44.4. Our data are comparable, but the median OS in our low group was shorter (114 days), possibly due to the smaller number of patients in this sub-group. In other studies, the impressively long OS may have been due to inclusion of lower grade gliomas [5]. Our study aligns with current studies that show preoperative PNI is not a significant independent predictor of OS in GBM patients [7, 15].

Postoperative PNI may be associated with PFS and OS, but we were unable to demonstrate this on multivariate analysis. The pathophysiology behind this is unclear. Mechanisms proposed in other cancer types may be applicable. The intrinsic relationship between malnutrition, hypoalbuminaemia, immunosuppression and inflammation may explain the pathophysiological basis underlying PNI as a prognostic or predictive factor. Inflammation leads to hypoalbuminaemia by reducing its half-life and synthesis and increases capillary permeability resulting in the extravasation of albumin into the extracellular space [19, 20]. Consequently, there is a decreased antioxidant effect against common cancer antigens and the provision of extra substrates as building blocks for aberrant cellular proliferation [20]. Inflammation causes immunosuppression by inhibiting lymphocytes which are essential effector cells in anti-tumour immunity by inhibiting cancer cell proliferation, invasion and migration [5]. Specifically, T cell activation and proliferation are inhibited, major histocompatibility complex expression is downregulated, and tumour-associated macrophages are skewed towards an immunosuppressive phenotype [21]. Shibutani et al. [22] demonstrated that OS was significantly worse in colorectal cancer patients with PNI < 45 (*p* = 0.0005), suggesting that this was due to the correlation between postoperative PNI and intraoperative blood loss. However, they could not exclude the confounding effect of postoperative complications on hypoalbuminaemia secondary to a systemic inflammatory response or long-term fasting. Since postoperative complications were part of our exclusion criteria, this mechanism is unlikely to have affected our conclusions. Zhang et al. [23] found that postoperative PNI < 53.05 was an independent predictor of both PFS (*p* = 0.007) and OS (*p* = 0.004) for patients with hepatocellular carcinoma, meeting Milan criteria and hypersplenism. This observation may reflect a shifting equilibrium between immune and inflammatory responses following surgical resection and the restoration of normal splenic immune function post-tumour removal [23].

The biological foundation underpinning the prognostic significance of NLR is not fully understood. Chronic systemic inflammation and subsequent myelopoiesis manifesting as neutrophilia is a hallmark of cancer. Neutrophilia suppresses the anti-tumour immune response through the marginalization and apoptosis of lymphocytes involved in the adaptive immune response [12]. Inversely, elevated NLR is also associated with an increase in cytokines involved in the innate immune response [24]. Simultaneously, neutrophilia perpetuates tumorigenesis by promoting metastasis, angiogenesis and leakage of tumour and endothelial cells into circulation [25].

In our study, preoperative NLR was not significantly associated with PFS or OS on univariate and multivariate analysis. This observation may not be dependent on the actual NLR cut-off value, because others have shown that cut-offs ranging from 2.5–4 did not result in a positive association [1]. This also lends support to our decision to use average values from the literature to define cut-offs, instead of developing our own, since the variation in cut-off values is most likely due to patient selection resulting in different cohort outcomes. Lopes et al. [10] found that although preoperative NLR was not associated with OS (*p* = 0.868), it was an independent prognostic marker for PFS (*p* = 0.032). This may be explained by their inclusion of different adjuvant treatment regimens, whereas we found no significant associations between preoperative NLR, PFS and non-conventional adjuvant chemotherapy (either 2^nd^ line chemotherapy post-progression or regimens not including TMZ). The different observations amongst studies may be due to the exclusion of patients with full blood counts performed following dexamethasone administration (this patient subgroup was included in our real-world study, as most patients received dexamethasone). Although a recent systematic review and meta-analysis of 2275 patients and 16 articles showed that preoperative NLR was a predictor of OS in GBM patients, confounding factors such as preoperative physical condition, comorbidities, infections, and concomitant medications have not been taken into account [26], unlike in our study. We found that a low vs high postoperative NLR was significantly associated with improved OS, yet, when other confounders were taken into consideration on multivariate analysis, this statistical significance diminished. To our knowledge, only two studies to date have analyzed postoperative NLR and survival in GBM patients. Maas et al. [27] found that a postoperative NLR > 4 was significantly correlated with adverse OS (*p* = 0.026), but this disappeared on multivariate analysis (*p* = 0.616). Wiencke et al. [28], utilizing methylation-derived cell composition estimates to calculate NLR (mdNLR) from blood samples taken a median of 100 days following histological GBM diagnosis, found that mdNLR > 4 conferred a worse OS (669 vs 1582 days, *p* = 0.02) and that mdNLR was significantly associated with survival time independent of therapy (*p* = 0.049). The longer survival times for both mdNLR groups suggests that patient selection may be the reason for the different outcomes noted in our study.

Whilst both preoperative and postoperative PNI and NLR were not directly linked with survival on multivariate analysis in this study, these inflammatory markers may play a role in the decision to implement emerging GBM adjuvant therapies. Following standard GBM treatment, the addition of tumour treating fields therapy to maintenance TMZ chemotherapy has been demonstrated to increase OS by 4.9 months via a variety of molecular mechanisms including an increase in the infiltration of cytotoxic T cells [29, 30]. Thus, a low PNI or high NLR highlights a subset of patients with relative lymphocyte deficit and poorer anti-tumour immunity who may benefit from this modality. The ketogenic diet (KD), results in a glucose-deprived, nutritional ketosis state [31]. This has been demonstrated to slow malignant glioma growth and improve survival when combined with anti-angiogenic or anti-glutamine therapy in mouse models [32, 33]. Although the evidence regarding GBM outcomes and KD in human adults is limited to single case reports, randomised controlled trials are currently underway. Until the results of these are available, the decision to clinically implement the KD must be weighed against the risk of rapid weight loss. Therefore, PNI has potential as a stratification tool to identify patients where the KD may present more risk (typically patients with a low PNI due to their already nutritionally poor state).

We found that postoperative ECOG score (1 vs 0) was statistically significant for PFS on multivariate analysis. The majority of GBM survival studies evaluate only preoperative ECOG. However, Gately et al. [34] and Saether et al. [35] found that increased postoperative ECOG score was significantly associated with poorer OS (*p* = 0.001 and *p* < 0.001, respectively). Thus, it is evident that a relationship exists between postoperative ECOG and GBM survival and future research is warranted. *IDH* mutation was an independent positive prognostic biomarker for PFS in our study which is in concordance with most existing literature [36]. We also found that dexamethasone was an independent determinant of shorter PFS. This phenomenon has been well noted in literature with studies suggesting that dexamethasone is administered in patients with more aggressive disease, it may have antagonistic effects on TMZ, has immunosuppressive effects, and may have a propensity to cause epigenetic changes on proliferative, invasive and angiogenic gene expression [37–39]. Curiously, postoperative dexamethasone administration differed significantly between low and high postoperative NLR and PNI groups which may have potentially confounded the survival results of our study.

Greater EOR was the best independent prognostic marker for improved OS in GBM patients in this study. EOR depends on tumour size, location, preoperative neurological status and surgeon’s experience [40]. Increased EOR is complicated by greater potential for surgically-acquired motor and language deficits. Hence, surgical aims are to maximize the field of resection whilst minimizing neurological complications [41]. Whilst the positive correlation between EOR and OS has been previously demonstrated, these studies used divergent categorical definitions of GTR (90–100%) and STR (0–99%) [41, 42]. Only a small number of these studies have volumetrically defined minimal percentage cut-off values for tumour resection conferring a survival benefit, with results ranging from 70–98% [41–43]. Those studies showing no association between EOR and survival were limited by the confounding effect of tumour proximity to the subventricular zone, intra and interobserver variability, prolonged follow-up MR images and lack of inclusion of GBM radiological features [44, 45]. To truly determine the correlation between EOR and survival, future studies should be prospective in nature with standardized qualitative or volumetric definitions for EOR.

Having had any previous surgery was the second most statistically independent predictor of adverse OS in GBM patients in our study. To date, patients’ surgical histories (unrelated to GBM) have not been included in GBM survival analyses. Although contentious, studies have proposed that surgery may result in long-term neurotoxicity and neurodegeneration [46]. Specifically, rodent studies have found that exposure to common surgical sedative and anaesthetic agents resulted in cell and synapse loss, clinically presenting as persistent cognitive deficits [47]. Other research has proposed that the surgical stress response independently propagates the pathophysiological mechanisms of Alzheimer’s disease [48]. There may also be a link between anaesthetic type and increased tumour retention and metastases as well as suppression of anti-cancer immunity [49].

### Limitations

This was a single-centre retrospective study with a small cohort size (n = 87). We utilized the mean of pre-established cut-off values in literature for NLR and PNI whilst other studies determined theirs through receiver operating characteristic analysis^6, 8^ X-tile software [5, 15], a linear mixed model [11] or classification and regression trees [7], yielding variable results. Some studies tested a range of successive cut-off values that were pre-established within literature and utilized the cut-off with the most significant *p* value for OS [3, 4, 10, 16]. Our study had a heterogeneous treatment population; patients that declined treatment or were too ill to undergo adjuvant therapy were included. Finally, we did not exclude patients on dexamethasone at data collection despite its potential confounding effects, because this comprised the majority of our cohort and was reflective of a true clinical setting.

## Conclusion

EOR was the most statistically significant independent prognostic biomarker for OS, closely followed by having had any previous surgery. Postoperative ECOG, *IDH* mutation and daily dexamethasone were significant independent predictors for PFS. Postoperative PNI was associated with PFS and OS but this did not remain significant on multivariate analysis. Despite this study’s limitations, these findings highlighted the influence of systemic inflammation on GBM survival outcomes. Further studies are required to validate the relationship between postoperative PNI and survival and to investigate mechanisms of systemic inflammation on the brain and GBM tumour microenvironment. Once this occurs, serum inflammatory markers may influence treatment decisions, especially in situations when EOR has been minimal and may pave the way for implementation of immunotherapy in future GBM treatment regimens.

## Supporting information

S1 Table Univariate and multivariate analysis for PFS and OS. (PDF)
