# Supplementary material for: Comparison of neutrophil to lymphocyte ratio and prognostic nutritional index with other clinical and molecular biomarkers for prediction of glioblastoma multiforme outcome
Source: PLoS One. 2021 Jun 17;16(6):e0252614. doi: 10.1371/journal.pone.0252614 (PMC8211244; doi:10.1371/journal.pone.0252614)
Supplement: S1 Table — (PDF) [file pone.0252614.s001.pdf]

**S1 Table.** Univariate and multivariate analyses for PFS and OS

| | Univariate analysis | | | | | | Multivariate analysis | | | | | |
|---|---|---|---|---|---|---|---|---|---|---|---|---|
| | HR | | 95% CI | | P value* | | HR | | 95% CI | | P value | |
| **Variables** | **PFS** | **OS** | **PFS** | **OS** | **PFS** | **OS** | **PFS** | **OS†** | **PFS** | **OS†** | **PFS** | **OS†** |
| Type | 0.268 | 0.267 | 0.037 – 1.967 | 0.035 – 2.038 | 0.196 | 0.203 | - | - | - | - | - | - |
| Gender | 0.532 | 0.670 | 0.315 – 0.900 | 0.408 – 1.098 | **0.019** | 0.112 | 0.722 | - | 0.324 – 1.611 | - | 0.427 | - |
| BMI | 0.968 | 0.960 | 0.931 – 1.006 | 0.925 – 0.996 | 0.098 | **0.028** | - | - | - | - | - | 0.269 |
| IDH mutation | 0.186 | 0.037 | 0.045 – 0.770 | 0.001 – 1.008 | **0.020** | 0.051 | 0.195 | - | 0.039 – 0.970 | - | **0.046** | - |
| MGMT methylation | 0.656 | 0.553 | 0.322 – 1.338 | 0.258 – 1.183 | 0.247 | 0.127 | - | - | - | - | - | - |
| EGFR mutation | | | | | | | | | | | | |
| EGFR mutation vs without | 1.444 | 2.205 | 0.531 – 3.927 | 0.778 – 6.255 | 0.471 | 0.137 | - | - | - | - | - | - |
| EGFRvIII vs without | 1.050 | 0.187 | 0.207 – 5.330 | 0.022 – 1.583 | 0.953 | 0.124 | - | - | - | - | - | - |
| Ki67 | 0.995 | 0.994 | 0.983 – 1.008 | 0.981 – 1.008 | 0.474 | 0.406 | - | - | - | - | - | - |
| GFAP mutation | 0.737 | 0.817 | 0.099 – 5.462 | 0.111 – 6.017 | 0.765 | 0.843 | - | - | - | - | - | - |
| Temozolomide | 1.182 | 0.425 | 0.767 – 1.823 | 0.177 – 1.024 | 0.449 | 0.056 | - | - | - | - | - | - |
| Temozolomide average dose | 0.996 | 0.995 | 0.992 – 0.999 | 0.991 – 0.999 | **0.020** | **0.018** | 0.999 | - | 0.994 – 1.004 | - | 0.690 | 0.586 |
| Radiotherapy | 0.094 | 0.101 | 0.038 – 0.228 | 0.050 – 0.203 | **<0.001** | **<0.001** | 0.523 | - | 0.062 – 4.425 | - | 0.552 | 0.456 |
| Radiotherapy total dose | 0.995 | 0.972 | 0.971 – 1.020 | 0.948 – 0.997 | 0.717 | **0.029** | - | - | - | - | - | - |
| Received another chemotherapy | 0.820 | 0.742 | 0.501 – 1.343 | 0.454 – 1.211 | 0.431 | 0.233 | - | - | - | - | - | - |
| Dexamethasone | 2.643 | 2.536 | 1.419 – 4.922 | 1.336 – 4.816 | **0.002** | **0.004** | 3.599 | - | 1.197 – 10.819 | - | **0.023** | 0.231 |
| Anti-convulsants | 1.153 | 1.278 | 0.691 – 1.926 | 0.769 – 2.123 | 0.585 | 0.344 | - | - | - | - | - | - |
| Neurological conditions | 0.687 | 1.015 | 0.275 – 1.716 | 0.461 – 2.234 | 0.422 | 0.970 | - | - | - | - | - | - |
| Dyslipidaemia/hypercholesterolaemia | 0.966 | 0.906 | 0.567 – 1.648 | 0.524 – 1.564 | 0.900 | 0.722 | - | - | - | - | - | - |
| Type II diabetes | 0.888 | 0.761 | 0.474 – 1.664 | 0.405 – 1.430 | 0.711 | 0.396 | - | - | - | - | - | - |
| Hypertension | 0.955 | 0.812 | 0.577 – 1.580 | 0.489 – 1.348 | 0.857 | 0.420 | - | - | - | - | - | - |
| Depression | 2.388 | 1.923 | 0.994 – 5.737 | 0.820 – 4.508 | 0.052 | 0.133 | - | - | - | - | - | - |
| Other cancer | 2.100 | 2.217 | 0.948 – 4.652 | 0.988 – 4.977 | 0.067 | 0.054 | - | - | - | - | - | - |
| Previous surgery | 1.838 | 2.065 | 1.045 – 3.231 | 1.192 – 3.575 | **0.035** | **0.010** | 1.948 | 2.825 | 0.874 – 4.345 | 1.234 – 6.466 | 0.103 | **0.014** |
| Age at diagnosis | 1.019 | 1.026 | 1.002 – 1.036 | 1.008 – 1.045 | **0.029** | **0.006** | 0.987 | - | 0.959 – 1.016 | - | 0.378 | 0.530 |
| EOR | | | | | | | | | | | | |
| STR vs biopsy | 0.252 | 0.288 | 0.112 – 0.567 | 0.146 – 0.567 | **0.001** | **<0.001** | 0.408 | 0.205 | 0.102 – 1.628 | 0.061 – 0.697 | 0.204 | **0.011** |
| GTR vs biopsy | 0.111 | 0.130 | 0.046 – 0.267 | 0.061 – 0.280 | **<0.001** | **<0.001** | 0.325 | 0.145 | 0.071 – 1.490 | 0.039 – 0.539 | 0.148 | **0.004** |
| Preoperative | | | | | | | | | | | | |
| ECOG | | | | | | | | | | | | |
| 1 vs 0 | 1.039 | 0.810 | 0.616 – 1.753 | 0.484 – 1.357 | 0.886 | 0.423 | - | - | - | - | - | - |
| 2 vs 0 | 1.488 | 1.406 | 0.510 – 4.342 | 0.534 – 3.701 | 0.467 | 0.490 | - | - | - | - | - | - |
| 3 vs 0 | 1.341 | 1.399 | 0.179 – 10.060 | 0.187 – 10.441 | 0.775 | 0.744 | - | - | - | - | - | - |
| Dexamethasone | 1.449 | 1.282 | 0.852 – 2.465 | 0.756 – 2.175 | 0.171 | 0.357 | - | - | - | - | - | - |
| NLR | 1.057 | 1.002 | 0.631 – 1.773 | 0.604 – 1.662 | 0.833 | 0.994 | - | - | - | - | - | - |
| PNI | 0.922 | 0.566 | 0.522 – 1.629 | 0.332 – 0.964 | 0.780 | **0.036** | - | - | - | - | - | 0.369 |
| Postoperative | | | | | | | | | | | | |
| ECOG | | | | | | | | | | | | |
| 1 vs 0 | 1.260 | 1.787 | 0.710 – 2.235 | 0.961 – 3.322 | 0.430 | 0.066 | 0.417 | - | 0.179 – 0.973 | - | **0.043** | 0.115 |
| 2 vs 0 | 1.739 | 3.057 | 0.845 – 3.578 | 1.522 – 6.141 | 0.133 | **0.002** | 0.645 | - | 0.211 – 1.975 | - | 0.443 | 0.797 |
| 3 vs 0 | 3.795 | 5.471 | 1.597 – 9.023 | 2.278 – 13.135 | **0.003** | **<0.001** | 0.900 | - | 0.198 – 4.090 | - | 0.892 | 0.056 |
| 4 vs 0 | 0.000 | 9.587 | 0.000 – 0.000 | 2.079 – 44.208 | 0.982 | **0.004** | - | - | - | - | - | 0.057 |
| Dexamethasone | 1.806 | 2.581 | 1.092 – 2.985 | 1.525 – 4.370 | **0.021** | **<0.001** | 0.968 | - | 0.477 – 1.965 | - | 0.928 | 0.140 |
| NLR | 1.395 | 1.832 | 0.783 – 2.484 | 1.054 – 3.185 | 0.258 | **0.032** | - | - | - | - | - | 0.179 |
| PNI | 0.501 | 0.436 | 0.296 – 0.846 | 0.259 – 0.735 | **0.010** | **0.002** | 0.804 | - | 0.390 – 1.659 | - | 0.555 | 0.151 |

BMI = body mass index, IDH = isocitrate dehydrogenase, MGMT = $O^6$methylguanine DNA methyltransferase, EGFR = epidermal growth factor receptor, GFAP = glial fibrillary acidic protein, EOR = extent of resection, STR = subtotal resection, GTR = gross total resection, ECOG = Eastern Cooperative Oncology Group, NLR = neutrophil to lymphocyte ratio, PNI = prognostic nutritional index

**P* values in bold are significant, †Results from multivariate analysis with forward selection process
